# Elevated Fasting Blood Glucose Levels Are Associated With Lower Cognitive Function, With a Threshold in Non-Diabetic Individuals: A Population-Based Study

**DOI:** 10.2188/jea.JE20180193

**Published:** 2020-03-05

**Authors:** Ziyi Liu, Maryam Zaid, Takashi Hisamatsu, Sachiko Tanaka, Akira Fujiyoshi, Naoko Miyagawa, Takahiro Ito, Aya Kadota, Ikuo Tooyama, Katsuyuki Miura, Hirotsugu Ueshima

**Affiliations:** 1 Molecular Neuroscience Research Center, Shiga University of Medical Science, Shiga, Japan; 2 Department of Public Health, Shiga University of Medical Science, Shiga, Japan; 3 Center for Epidemiologic Research in Asia, Shiga University of Medical Science, Shiga, Japan; 4 Department of Environmental Medicine and Public Health, Faculty of Medicine, Shimane University, Shimane, Japan; 5 Department of Medical Statistics, Shiga University of Medical Science, Shiga, Japan

**Keywords:** cognitive abilities screening instrument (CASI), cognitive function, diabetes mellitus, fasting blood glucose

## Abstract

**Background:**

Cognitive dysfunction has been recognized as a diabetes-related complication. Whether hyperglycemia or elevated fasting glucose are associated with cognitive decline remains controversial. We aimed to investigate the relationship between fasting glucose levels and cognitive function in diabetic and non-diabetic individuals.

**Methods:**

Participants were Japanese diabetic (*n* = 191) and non-diabetic (*n* = 616) men, aged 46–81 years, from 2010–2014. Blood samples were taken after a 12 h fast. The Cognitive Ability Screening Instrument (CASI), with a maximum score of 100, was used for cognitive assessment. Cognitive domains of CASI were also investigated. Fractional logit regression with covariate adjustment for potential confounders was used to model cross-sectional relationships between fasting blood glucose and CASI score.

**Results:**

For diabetic individuals, CASI score was 0.38 (95% confidence interval: 0.66–0.12) lower per 1 mmol/L higher fasting glucose level. Short-term memory domain also exhibited an inverse association. For non-diabetic individuals, a reverse U-shaped relationship was observed between fasting glucose and cognitive function, identifying a threshold for highest cognitive performance of 91.8 CASI score at 3.97–6.20 mmol/L (71.5–111.6 mg/dL) fasting glucose. Language ability domain displayed a similar relationship with fasting glucose.

**Conclusions:**

Elevated fasting glucose levels in diabetic men were associated with lower cognitive function, in which short-term memory was the main associated domain. Interestingly, in non-diabetic men, we identified a threshold for the inverse relationship of elevated fasting glucose with cognitive function. Contrastingly to diabetic men, language ability was the main associated cognitive domain among non-diabetic men.

## INTRODUCTION

Global prevalence of diabetes is projected to increase, and by 2030 around 366 million people worldwide are expected to be affected.^1^ Individuals with diabetes mellitus have increased risk of cognitive dysfunction.^2^^,^^3^ Studies have reported that diabetic patients have up to a 2.5-fold greater risk of dementia compared to non-diabetic individuals.^4^^,^^5^ As a diabetes-related complication, dementia has become a serious burden to Japanese society, healthcare, and economy.^6^

Diabetes is a multifactorial and complex disease that is associated with other metabolic and health complications.^7^^–^^9^ However, its underlying conditions, such as hyperglycemia and insulin resistance, are considered to be the link between diabetes and cognitive decline.^10^ Studies suggest that hyperglycemia-related increased oxidative stress and accumulation of advanced glycation end-products (AGEs) can lead to vascular and neurovascular damage.^11^^,^^12^ However, there are some studies that report little to no association between hyperglycemia and cognitive dysfunction in type II diabetes.^13^

There are fewer studies on fasting glucose levels and cognitive function in non-diabetic individuals.^14^ Moreover, conflicting results have been reported on whether elevated fasting glucose in non-diabetic individuals are associated with cognitive impairment.^15^^–^^18^ This has led to the speculation about a threshold for effects of glucose on cognitive function in non-diabetic individuals.^15^

We aimed to analyze the associations of fasting blood glucose with cognitive function and cognitive domains in non-diabetic and diabetic Japanese men.

## METHODS

### Study participants

The Shiga Epidemiological Study of Subclinical Atherosclerosis (SESSA) is a population-based study which recruited 40- to 79-year-old men, randomly selected in 2006–2008 (baseline), from the general population in Kusatsu City, Shiga, Japan (participation rate of 46%).^19^^–^^21^ Although SESSA is an observational cohort study with follow-up in 2010–2014, our present analysis only involves data from the follow-up because CASI score was not measured at baseline. A total of 853 men participated in the follow-up, a participation rate of 78% (see eFigure 1 for selection flow).

A questionnaire was completed by all participants. Data on years of education, medical history, use of medication (such as hypertension, hyperlipidemia, and diabetes medication), and other lifestyle characteristics were collected.

Each participant had undergone a physical examination involving measurements of height, weight, blood pressure, and other various physical attributes. Blood pressure was measured after 5 minutes of rest in a seated position using an automated sphygmomanometer (BP-8800; Omron Colin, Tokyo, Japan). An average of two blood pressure measurements was used.

Participants with a systolic blood pressure (SBP) of ≥140 mm Hg, a diastolic blood pressure (DBP) of ≥90 mm Hg, or the use of antihypertensive medication were characterized as having hypertension. Body mass index (BMI) was defined as weight divided by height squared (kg/m^2^). Current smokers were defined as individuals who smoked tobacco within the last 30 days. Current drinkers were defined as individuals who answered “yes” to the question “do you currently drink alcohol?”.

After excluding participants with self-reported Parkinson’s disease (*n* = 4) and with missing information on fasting blood glucose values or CASI score (*n* = 43), a total of 807 men aged 46–81 years remained in the current analysis. All participants provided written and informed consent. This study complies with The Code of Ethics of the World Medical Association (Declaration of Helsinki) and was approved by the Institutional Review Board of Shiga University of Medical Science, Otsu, Japan.

### Blood samples, glucose, and diabetes

Fasting blood samples were taken from participants after a 12 hour fast. Standard lipids were measured using enzymatic techniques. Lipid measurements were standardized according to the guidelines from the Center for Disease Control and Prevention/Cholesterol Reference Method Laboratory Network.^22^ Sodium fluoride-treated plasma was used to measure fasting blood glucose by hexokinase/glucose-6-phosphate dehydrogenase enzymatic assay. Glycated hemoglobin A1c (HbA1c) was measured by latex agglutination assay according to National Glycohemoglobin Standardization Program (NGSP) or Japan Diabetes Society (JDS) protocol. The number of participants who had HbA1c measured by NGSP or JDS was 194 and 743, respectively. JDS values were converted to NGSP values using the following equation^23^: NGSP (%) = 1.02 × JDS value (%) + 0.25. International Federation of Clinical Chemistry (IFCC) values for HbA1c can be calculated from NGSP values using the following equation: IFCC (mmol/mol) = 10.93 × NGSP (%) − 23.50. Diabetes was defined as taking diabetes medication, blood glucose to ≥6.99 mmol/L (≥126 mg/dL) or HbA1c ≥6.5% (NGSP).

### Cognitive Ability Screening Instrument

CASI was used to assess cognitive ability. CASI is a 15–20 minute cognitive assessment that was developed for cross-cultural use, which has also been found to closely estimate scores from other cognitive tests, such as Mini-Mental State Examination (MMSE).^24^ A trained technician used the Japanese version of CASI (CASI J-1.0) to assess cognitive function. In CASI, a total of nine cognitive domains were assessed (maximum points): attention (8 points), concentration/mental manipulation (10 points), orientation (18 points), short-term memory (12 points), long-term memory (10 points), language (10 points), visual construction (10 points), list-generating fluency (10 points), and abstraction/judgement (12 points). The scores in all domains were added to provide a total CASI score with a range of 0 to 100, with higher scores indicating better cognitive function.^24^

### Statistical analysis

All analyses were performed on non-diabetic and diabetic participants separately. Participant demographics were described according to diabetes status. Differences between non-diabetic and diabetic groups were assessed with student’s *t*-test (means) for continuous variables and χ^2^ test (proportions) for categorical variables.

Non-diabetic and diabetic participants were separated into groups according to deciles of fasting blood glucose. Analysis of covariance was used to calculate mean total CASI score, adjusted for age, total years of education, and (for diabetic only) use of diabetes medication for all decile groups.

Fractional logit regression was used to model the relationship between CASI and fasting blood glucose as total CASI score has minimum and maximum limits (0 and 100). Thus, to ensure predicted values fall between these limits, CASI was modeled as a proportion by the total maximum score (CASI/100) in the fractional logit model. Similarly, CASI domain scores were divided by their allotted maximum points (eg, short-term memory was divided by 8). Both linear and quadratic relationships between glucose and CASI were explored in non-diabetic and diabetic individuals. The linear relationship between CASI and glucose was modeled (in its simplest form) as: CASI/100 = 1/(1 + e^−(a^*^x^*^+b)^), where *x* is glucose. For quadratic analyses, a similar equation was used, but instead with the negative exponent of *e* being a*x*^2^ + b*x* + c. Estimated glucose level at peak CASI score was obtained from the vertex equation (−b/2a). Bootstrap method, with 1,000 samples of data generated randomly from original data (with replacement), was used to identify 95% confidence limits of the glucose estimate at peak CASI score using 2.5 and 97.5 percentiles of estimated peak glucose.

Regression models were adjusted for the following covariates: (model 1) age and total years of education, (model 2) Model 1 + body mass index, systolic blood pressure, hypertension medication (yes/no), lipid medication (yes/no), and smoking status (yes/no). In diabetic individuals, all models were further adjusted for diabetic medication (yes/no). No interactions were evident for age as a continuous variable or as a categorical variable (<65 and ≥65 years old) with CASI and fasting blood glucose in either non-diabetic or diabetic individuals.

All analyses were performed using SAS version 9.4 (SAS Institute, Cary, NC, USA) and two-tailed *P*-values were reported.

## RESULTS

Of the total 807 Japanese male participants, 616 were non-diabetic and 191 were diabetic. Mean age of non-diabetic and diabetic participants was 67.6 and 69.8 years, respectively. Diabetic participants were older, had higher SBP and BMI, and had lower levels of total and HDL cholesterol. They were more likely to be current smokers and to take hypertension and lipid medication than non-diabetic participants (Table 1). Compared to non-diabetic participants, diabetic participants had a lower total CASI score and generally lower CASI domain scores, especially for concentration and short-term memory (Table 2). Non-diabetic participants had a mean fasting blood glucose level of 5.2 mmol/L and a range of 3.7–6.9 mmol/L. Diabetic participants had a mean glucose value of 7.1 mmol/L and a range of 2.5–14.9 mmol/L.

**Table 1.** Characteristics of men aged 46–81 years according to diabetes status in 2010–2014, Shiga, Japan (*n* = 807)

Characteristic	Non-Diabetic (*n* = 616)		Diabetic (*n* = 191)		*P*-value
Age, years	67.6	(8.4)	69.8	(6.9)	<0.001
Years of education	12.8	(2.4)	12.7	(2.4)	0.428
SBP, mm Hg	131	(17)	134	(16)	0.039
Body mass index, kg/m^2^	23.1	(2.8)	24.2	(3.4)	<0.001
Total cholesterol, mmol/L	5.2	(0.9)	5.1	(0.9)	0.033
HDL-cholesterol, mmol/L	1.6	(0.4)	1.4	(0.4)	<0.001
Smoking amount, cig/day	3.0	(7.1)	4.5	(9.2)	0.036
Current smoker, %	19.7		26.2		0.055
Current drinker, %	80.5		75.4		0.129
Hypertension medication, %	35.0		59.2		<0.001
Diabetes medication, %	0.0		69.6		<0.001
Lipid medication, %	16.9		40.8		<0.001

HDL, high-density lipoprotein; SBP, systolic blood pressure.

Data are presented as mean (standard deviation), or as percentages, as indicated.

*P*-value for the difference between non-diabetic and diabetic group was derived from student's *t*-test for comparison of means or χ2 test for comparison of percentages.

**Table 2.** Fasting blood glucose, HbA1c, and total and domain CASI scores in non-diabetic and diabetic men

Measure	Non-Diabetic (*n* = 616)			Diabetic (*n* = 191)			*P*-value
	
	Mean (SD)	Median (IQR)	Range	Mean (SD)	Median (IQR)	Range	
Glucose, mmol/L	5.2 (0.5)	5.2 (4.9–5.6)	3.7–6.9	7.1 (1.8)	7.0 (6.1–7.6)	2.5–14.9	<0.001
HbA1c, %^a^	5.6 (0.4)	5.6 (5.4–5.9)	4.3–6.4	7.0 (1.0)	6.8 (6.5–7.4)	5.0–12.9	<0.001
CASI score (/100 points)	91.1 (5.7)	92 (88–95)	66–100	90.0 (6.2)	91 (87–94)	63–100	0.024
Attention (/8 points)	7.0 (1.0)	7 (6–8)	3–8	6.8 (1.0)	7 (6–8)	5–8	0.145
Concentration (/10 points)	9.0 (1.4)	10 (8–10)	1–10	8.7 (1.7)	10 (8–10)	1–10	0.017
Orientation (/18 points)	17.7 (0.9)	18 (18–18)	7–18	17.7 (1.0)	18 (18–18)	9–18	0.654
Short-term memory (/12 points)	9.5 (2.1)	10 (8–11)	1–12	9.1 (2.2)	10 (8–11)	3–12	0.033
Long-term memory (/10 points)	10.0 (0.3)	10 (10–10)	8–10	9.9 (0.4)	10 (10–10)	7–10	0.441
Language (/10 points)	9.8 (0.5)	10 (10–10)	7–10	9.8 (0.7)	10 (10–10)	5–10	0.232
Visual construction (/10 points)	9.8 (0.8)	10 (10–10)	0–10	9.8 (0.4)	10 (10–10)	8–10	0.594
List-generating fluency (/10 points)	8.8 (1.6)	10 (8–10)	0–10	8.8 (1.6)	9 (8–10)	2–10	0.752
Abstraction (/12 points)	9.5 (1.8)	10 (9–11)	3–12	9.4 (1.8)	10 (8–11)	4–12	0.392

CASI, Cognitive Ability Screening Instrument; HbA1c, glycated hemoglobin A1c; IQR, interquartile range; SD, standard deviation.

The maximum points allotted for CASI and its domains are indicated (eg /10 points).

*P*-value was derived from student’s *t*-test and is for the difference between non-diabetic and diabetic groups.

For conversion of glucose from mmol/L to mg/dL, multiply by 18.

aNGSP HbA1c levels. To convert to IFCC HbA1c values: IFCC (mmol/mol) = 10.93 × NGSP (%) − 23.50.

### Non-diabetic individuals

**Figure 1.**
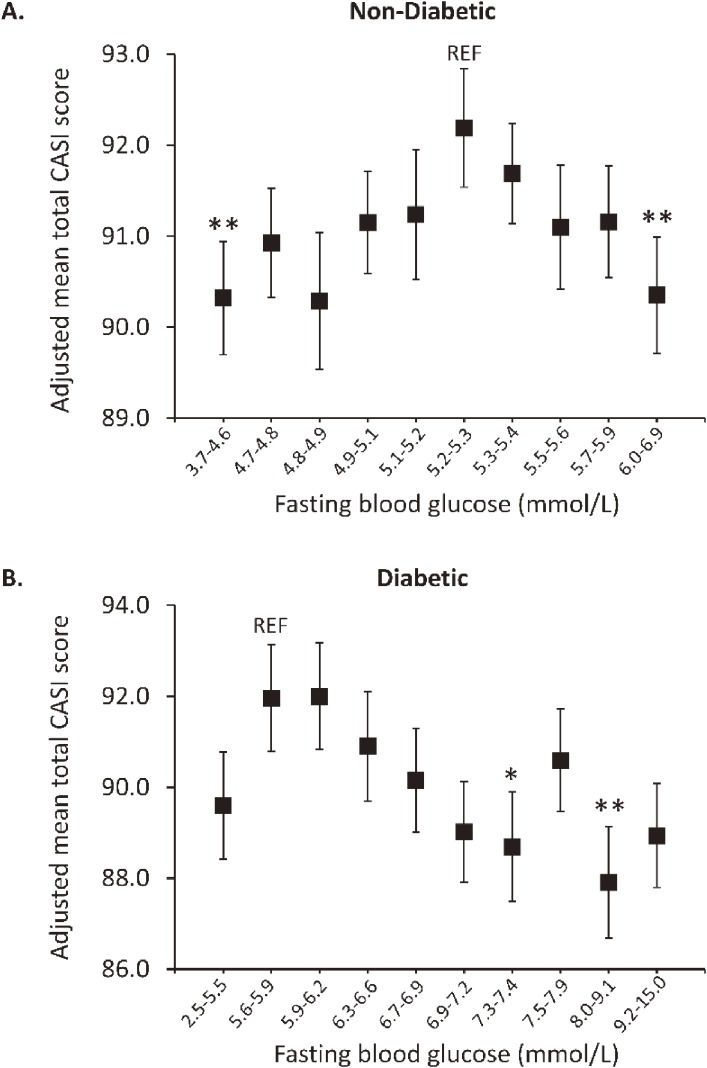
Adjusted mean total CASI scores by deciles of glucose in A) non-diabetic and B) diabetic men. Data presented are means with standard errors (error bars). Means were adjusted for age, total years of education, and (for diabetics only) diabetic medication. Mean CASI scores between glucose deciles were calculated and compared using analysis of covariance. CASI, Cognitive Ability Screening Instrument; REF, reference group. ^*^P = 0.05, ^**^P < 0.05

According to decile analysis, mean CASI scores adjusted for age and total years of education appear to have a curved relationship with fasting blood glucose (Figure 1). Nevertheless, both linear and quadratic relationships were investigated between glucose and CASI. No linear relationship was identified in non-diabetic individuals between glucose and CASI, even after adjustment for potential confounders and effect modifiers (data not shown). However, a reverse U-shaped relationship (ie, having a negative coefficient for the quadratic term) was found (Table 3). The vertex (ie, peak CASI score) of the quadratic function was identified as having an estimated 95% confidence range for glucose of 4.42 to 6.20 mmol/L (79.6 to 111.6 mg/dL) and highest CASI score of 91.8 with adjustments for age and total years of education (model 1). Further adjustment for BMI, SBP, hypertension medication, lipid medication, and smoking status (model 2) resulted in a similar range of 3.97 to 6.20 mmol/L (71.5 to 111.6 mg/dL) and highest CASI score of 91.8. With regard to CASI domains, only language ability domain had a reverse U-shaped relationship with glucose (Table 3), whereas other CASI domains did not (eTable 1).

**Table 3.** Estimated fasting blood glucose range associated with the highest total and domain CASI scores from the reverse U-shaped relationship in non-diabetic men

	Score	Highest score estimate	Glucose (mmol/L)	*P*-value
Model 1	Total CASI	91.8	4.42–6.20	0.042
	Language	9.9	4.53–5.79	0.007
Model 2	Total CASI	91.8	3.97–6.20	0.044
	Language	9.9	4.66–5.91	0.007

CASI, Cognitive Ability Screening Instrument.

Model 1 was adjusted for age and total years of education.

Model 2 was adjusted for age, total years of education, body mass index, systolic blood pressure, hypertension medication (yes/no), lipid medication (yes/no), and smoking status (yes/no).

*P*-value of quadratic coefficient (glucose*glucose), signifying a significant quadratic or U-shaped relationship, is displayed.

Estimated glucose range of peak CASI score was derived from the 95% confidence glucose values of the vertex of the quadratic function obtained from bootstrap sampling method (1000). Other CASI domains had non-significant relationships with glucose (see eTable 1).

### Diabetic individuals

Mean CASI scores adjusted for age, total years of education, and diabetic medication appear to have an inverse linear relationship with deciles of fasting blood glucose (Figure 1). No quadratic relationship was evident between glucose and CASI in diabetic individuals (data not shown). However, we observed a significant inverse linear relationship between glucose and CASI (Table 4), with adjustments made for age, total years of education, and diabetes medication (model 1). This relationship was maintained after further adjustments for BMI, SBP, hypertension medication, lipid medication, and smoking status (model 2). An inverse linear relationship of glucose was only evident with short-term memory CASI domain (Table 4) and not with other domains (eTable 2).

**Table 4.** Estimated difference in total and domain CASI scores per 1 mmol/L higher fasting blood glucose from the linear relationship in diabetic men

	Score	ΔCASI	95% CI	*P*-value
Model 1	Total CASI	−0.41	−0.70 to −0.17	0.038
	Short-term memory	−0.17	−0.24 to −0.05	0.022
Model 2	Total CASI	−0.38	−0.66 to −0.12	0.050
	Short-term memory	−0.16	−0.23 to −0.04	0.034

ΔCASI, difference in Cognitive Ability Screening Instrument; CI, confidence interval.

Model 1 was adjusted for age, total years of education and diabetic medication (yes/no).

Model 2 was adjusted for age, total years of education, diabetic medication (yes/no), body mass index, systolic blood pressure, hypertension medication (yes/no), lipid medication (yes/no), and smoking status (yes/no).

Other CASI domains had non-significant relationships with glucose (see eTable 2).

## DISCUSSION

### Summary

In both non-diabetic and diabetic men, we identified significant relationships of fasting blood glucose with total CASI score. In non-diabetic men, this was a reverse U-shaped relationship, suggesting a threshold at a glucose range of 3.97 to 6.20 mmol/L and language ability was a significant domain in this relationship. Whereas in diabetic men, an inverse linear relationship between fasting glucose and CASI was observed and short-term memory was a significant domain.

### Non-diabetic individuals

In non-diabetic individuals, we identified a range of glucose that is associated with highest CASI score. The reverse U-shaped relationship between glucose and CASI in non-diabetic individuals involved a peak CASI score within a 95% confidence glucose range of 4.42 to 6.20 mmol/L (79.6 mg/dL to 111.6 mg/dL) in a model adjusted for age and total years of education (model 1). With further adjustments for other important covariates (model 2), this range broadened at the lower extremity to become 3.97 to 6.20 mmol/L. The higher extreme glucose value of the range is very close to the cut-off for impaired fasting glucose (≥6.1 mmol/L or 110 mg/dL) recommended by WHO.^25^ Our study suggests presence of a threshold for the relationship between glucose and cognitive function and that impaired fasting glucose may be associated with cognitive impairment. There were limited number of participants in our study with glucose values near the lower extremity of the range. Only two participants in our study had glucose levels of less than 3.97 mmol/L. The lower extreme value of the range may not be conclusive and this was evident as this value was susceptible to change with further covariate adjustment, unlike the higher extreme value. Moreover, as participants were given the CASI test at a pre-prandial state, lack of food intake may have affected participants’ test performance,^26^ especially for those with low fasting blood glucose levels. Thus, although our study shows a reverse U-shaped relationship between cognitive function and fasting blood glucose, we cannot conclude that individuals with lower fasting blood glucose have lower cognitive ability.

There are limited studies on the relationship of blood glucose with cognitive function in non-diabetic individuals. However, in a community-based cohort,^27^ individuals with borderline diabetes had a higher 5-year hazard ratio of dementia and Alzheimer’s disease compared to those without borderline diabetes. Crane et al have also reported that in non-diabetic individuals, those with higher calculated average glucose levels in the preceding 5 years were at increased risk of dementia.^14^ The findings in these two studies suggest that even in individuals without diabetes, long-term exposure to high glucose levels leads to cognitive decline. In contrast, a study by Euser et al^15^ found no association between baseline fasting blood glucose levels and changes in cognitive function in non-diabetic individuals. However, this study has only assessed linear associations of baseline fasting glucose with cognitive test score and its changes over time in non-diabetic individuals. Interestingly, cross-sectional analysis of baseline data involving graphs depicting mean cognitive test score among quintiles of fasting glucose levels suggested a reverse U-shaped association, similar to our findings.

### Diabetic individuals

Although most studies on the relationship between glycemia and cognitive function have focused on diabetic individuals, inconsistent results were reported.^28^ Many studies, including a cohort study from Japan, have reported that people with diabetes have a greater risk of cognitive decline, dementia and Alzheimer’s disease.^8^^,^^28^^–^^31^ However, studies on the spectrum of glucose levels with cognitive function in diabetic individuals are rare. Crane et al has found that higher calculated average glucose levels in diabetic individuals were related to an increased risk of dementia.^14^ With regard to hypoglycemia, diabetic patients with hypoglycemia-associated hospitalizations were found to be at two-fold increased risk of developing dementia.^32^ Yet, we could not investigate such a relationship with cognitive function, as only four diabetic individuals had fasting blood glucose levels of <3.9 mmol/L (<70 mg/dL). However, we found that the first blood glucose decile group had a discernable lower mean CASI score compared to the adjacent groups (Figure 1B).

### CASI and its domains

CASI is a cross-cultural cognitive test that provides a quantitative assessment of various components of cognitive function and was designed to screen for dementia, monitor disease progression and to provide profiles of cognitive impairment.^24^ Importantly, dementia patients were found to have lower mean CASI compared to control subjects.^24^ Short-term memory was identified as one of the most sensitive CASI domains for distinguishing dementia patients from controls.^24^ In our results, glucose had an inverse relationship with short-term memory in diabetic individuals. Also, short-term memory was one of the two domains in which participants with diabetes obtained significantly lower scores than participants without diabetes. In participants without diabetes, rather than memory, language ability had a reverse U-shaped relationship with fasting blood glucose. It is not known whether low scores in memory or language in cognitive tests are precursors of true dementia or whether they represent other processes. Also, the early stages of different types of dementia can be substantially different from one another. Short-term memory is predominantly a hippocampal function,^33^ while language ability is a function of the temporal cortex and the inferior frontal cortex.^34^ However, the brain functions as an integrated network and thus labelling areas of the brain as focal areas of cognitive test components should be done so cautiously. Regardless, the different domains of CASI that are associated with high fasting blood glucose in non-diabetic and diabetic individuals suggests that blood glucose may affect cognitive function in these individuals using different pathways, possibly producing different long-term outcomes.

### Strengths and limitations

There are several limitations to our study. First, our study is cross-sectional and only provides a snapshot of the relationship between glucose and CASI. Also, we only present data on men and, thus, it is unclear whether these findings can be replicated in women. Moreover, residual confounding by other factors not considered (such as history of stroke, exercise, sleep apnea, and depression) cannot be ruled out.

Our study has some important strengths. First, we assessed a continuous variable of cognitive function over a spectrum of fasting blood glucose levels in both non-diabetic and diabetic individuals. Other studies have used calculated average glucose levels^14^ or have not done so in both non-diabetic and diabetic individuals.^15^ Second, we were able to identify a relationship with glucose prior to onset of dementia or Alzheimer’s disease in generally healthy individuals. Third, we identified different types of relationships of fasting blood glucose with cognitive function by diabetes status and found a threshold of fasting blood glucose for highest cognitive performance in individuals without diabetes. Finally, we identified specific domains of cognitive function that may be related to cognitive impairment in non-diabetic and diabetic individuals.

### Conclusion

We have identified relationships of fasting blood glucose and cognitive function in non-diabetic and diabetic men. A reverse U-shaped relationship was observed in non-diabetic men and we identified the threshold of fasting blood glucose for highest cognitive performance at a cut-off of <6.2 mmol/L. Language ability score also showed a similar trend. An inverse linear relationship was found in diabetic men, with short-term memory being a significant domain. For greater insight into the plausible causal relationship between blood glucose and cognitive ability, longitudinal observational studies are needed, especially in non-diabetic individuals.

## APPENDIX A. SUPPLEMENTARY DATA

eTable 1. Estimated logit coefficients of the reverse u-shaped relationship of total and domain CASI scores per 1 mmol/L higher glucose in non-diabetic men (*n* = 616)

eTable 2. Estimated logit coefficients of the linear relationship of total and domain CASI scores per 1 mmol/L higher glucose in diabetic men (*n* = 191)

eFigure 1. SESSA study design and study flow chart. Participating men in this study were those from the follow-up and only their data from the follow-up were used. ^*^For exclusion criteria, 1 person has overlapping exclusion criteria, having both self-reported Parkinson’s Disease and missing information.

## APPENDIX B.

### SESSA Research Group Members

Chairperson: Ueshima Hirotsugu (Center for Epidemiologic Research in Asia, Shiga University of Medical Science). Co-chairperson: Katsuyuki Miura (Department of Public Health, Shiga University of Medical Science). Research members: Minoru Horie (Department of Cardiovascular and Respiratory Medicine, Shiga University of Medical Science), Takashi Yamamoto (Department of Cardiovascular and Respiratory Medicine, Shiga University of Medical Science), Hideki Hayashi (Department of Cardiovascular and Respiratory Medicine, Shiga University of Medical Science), Yasutaka Nakano (Department of Cardiovascular and Respiratory Medicine, Shiga University of Medical Science), Emiko Ogawa (Health Administration Center, Shiga University of Medical Science), Hiroshi Maegawa (Division of Endocrinology and Metabolism, Department of Medicine, Shiga University of Medical Science), Itsuko Miyazawa (Division of Endocrinology and Metabolism, Department of Medicine, Shiga University of Medical Science), Kiyoshi Murata (Department of Radiology, Shiga University of Medical Science), Kenichi Mitsunami (Vories Memorial Hospital), Kazuhiko Nozaki (Department of Neurosurgery, Shiga University of Medical Science), Ikuo Tooyama (Molecular Neuroscience Research Center, Shiga University of Medical Science), Akihiko Shiino (Molecular Neuroscience Research Center, Shiga University of Medical Science), Teruhiko Tsuru (Department of Urology, Shiga University of Medical Science), Hisakazu Ogita (Division of Medical Biochemistry, Department of Biochemistry and Molecular Biology, Shiga University of Medical Science), Akio Shimizu (Division of Medical Biochemistry, Department of Biochemistry and Molecular Biology, Shiga University of Medical Science), Naomi Miyamatsu (Department of Clinical Nursing, Shiga University of Medical Science), Toru Kita (Kyoto University), Takeshi Kimura (Department of Cardiovascular Medicine, Kyoto University), Yoshihiko Nishio (Department of Diabetes, Metabolism, and Endocrinology, Kagoshima University), Yasuyuki Nakamura (Department of Food Science and Human Nutrition, Faculty of Agriculture, Ryukoku University), Tomonori Okamura (Department of Preventive Medicine and Public Health, School of Medicine, Keio University), Akira Sekikawa (Department of Epidemiology, Graduate School of Public Health, University of Pittsburgh), Emma JM Barinas-Mitchell (Department of Epidemiology, Graduate School of Public Health, University of Pittsburgh), Daniel Edmundowicz (Department of Medicine, Section of Cardiology, School of Medicine, Temple University), Takayoshi Ohkubo (Department of Hygiene and Public Health Teikyo University School of Medicine), Atsushi Hozawa (Department of Preventive Medicine and Epidemiology, Tohoku Medical Megabank Organization, Tohoku University), Yoshitaka Murakami (Division of Medical Statistics, Department of Social Medicine, Toho University), Nagako Okuda (Department of Health and Nutrition, University of Human Arts and Sciences), Hisatomi Arima (Department of Preventive Medicine and Public Health, Faculty of Medicine, Fukuoka University), Yoshikuni Kita (Faculty of Nursing Science, Tsuruga Nursing University), Takashi Hisamatsu (Department of Environmental Medicine and Public Health, Faculty of Medicine, Shimane University), Aya Higashiyama (Research and Desvelopment Initiative Center, National Cerebral and Cardiovascular Center), Shinya Nagasawa (Department of Epidemiology and Public Health, Kanazawa Medical University), Koichiro Azuma (Institute for Integrated Sports Medicine, Keio University School of Medicine), Masahiko Yanagita (Faculty of Health and Sports Science, Doshisha University), Robert D. Abbott (Center for Epidemiologic Research in Asia, Shiga University of Medical Science), Akira Fujiyoshi (Department of Public Health, Shiga University of Medical Science), Aya Kadota (Center for Epidemiologic Research in Asia, Shiga University of Medical Science), Naoyuki Takashima (Department of Public Health, Shiga University of Medical Science), Seiko Ohno (Center for Epidemiologic Research in Asia, Shiga University of Medical Science), Takashi Kadowaki (Department of Public Health, Shiga University of Medical Science), Sayaka Kadowaki (Department of Public Health, Shiga University of Medical Science), Naoko Miyagawa (Department of Public Health, Shiga University of Medical Science), Keiko Kondo (Department of Public Health, Shiga University of Medical Science), Sayuki Torii (Department of Public Health; Department of Cardiovascular and Respiratory Medicine, Shiga University of Medical Science), Maryam Zaid (Center for Epidemiologic Research in Asia, Shiga University of Medical Science), Yoshino Saito (Department of Public Health, Shiga University of Medical Science), Sentaro Suzuki (Department of Public Health, Shiga University of Medical Science), Takahiro Ito (Department of Public Health, Shiga University of Medical Science), Atsushi Satoh (Department of Public Health, Shiga University of Medical Science), and Masahiro Yamazoe (Department of Cardiology, Tokyo Medical and Dental University).
