# Supplementary material for: Elevated Fasting Blood Glucose Levels Are Associated With Lower Cognitive Function, With a Threshold in Non-Diabetic Individuals: A Population-Based Study
Source: J Epidemiol. 2020 Mar 5;30(3):121–7. doi: 10.2188/jea.JE20180193 (PMC7025918; doi:10.2188/jea.JE20180193)
Supplement: Supplementary file 1 [file je-30-121-s001.pdf]

Supplementary Material

eTable 1. Estimated logit coefficients of the reverse u-shaped relationship of total and domain CASI scores per 1 mmol/L higher glucose in non-diabetic men (n=616)

Score	Coeff. *a*	*p*-value	Coeff. *b*	*p*-value
Total CASI	1.23	0.042	-0.11	0.042
Attention	1.63	0.148	-0.16	0.118
Language	7.00	0.009	-0.66	0.007
Long-term memory	7.30	0.124	-0.71	0.099
Short-term memory	1.46	0.138	-0.12	0.178
Concentration	0.09	0.953	-0.01	0.935
Orientation	-2.83	0.464	0.30	0.411
Visual	0.67	0.860	-0.12	0.730
Abstract	0.93	0.292	-0.09	0.283
Fluency	1.57	0.311	-0.14	0.316

CASI, Cognitive Abilities Screening Instrument; Coeff., coefficient.

CASI scores were modeled as score/total maximum points (i.e. Total CASI/100, attention /8, etc). Thus, the linear relationship between total CASI and glucose was modeled (in its simplest form) as: CASI/100 = 1 / (1 + $e^{-(ax + bx*x + c)}$). The coefficient (Coeff.) values displayed above are for the "*a*" and "*b*" values, respectively.

eTable 2. Estimated logit coefficients of the linear relationship of total and domain CASI scores per 1 mmol/L higher glucose in diabetic men (n=191)

Score	Coeff.	*p*-value
Total CASI	-0.05	0.038
Attention	-0.04	0.358
Language	-0.09	0.456
Long-term memory	-0.16	0.403
Short-term memory	-0.08	0.022
Concentration	-0.10	0.099
Orientation	-0.08	0.461
Visual	0.12	0.315
Abstract	0.01	0.808
Fluency	-0.06	0.282

CASI, Cognitive Abilities Screening Instrument; Coeff., coefficient.

Model was adjusted for age, years of education, and use of diabetic medication (yes/no).

CASI scores were modeled as score/total maximum points (i.e. Total CASI/100, attention /8). Thus, the linear relationship between total CASI and glucose was modeled (in its simplest form) as: $CASI/100 = 1 / (1 + e^{-(ax+b)})$. The coefficient (Coeff.) displayed above is the "a" value.

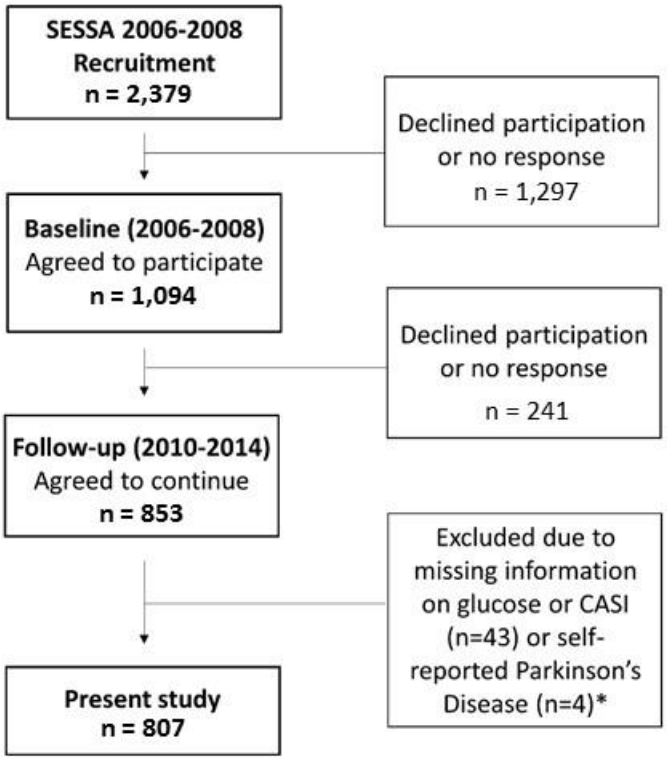

eFigure 1. SESSA study design and study flow chart. Participating men in this study were those from the follow-up and only their data from the follow-up were used. *For exclusion criteria, 1 person has overlapping exclusion criteria, having both self-reported Parkinson's Disease and missing information.
